# IL-13 Impairs Tight Junctions in Airway Epithelia

**DOI:** 10.3390/ijms20133222

**Published:** 2019-06-30

**Authors:** Hanna Schmidt, Peter Braubach, Carolin Schilpp, Robin Lochbaum, Kathrin Neuland, Kristin Thompson, Danny Jonigk, Manfred Frick, Paul Dietl, Oliver H. Wittekindt

**Affiliations:** 1 Institute of General Physiology, Ulm University, Albert-Einstein-Allee 11, 89081 Ulm, Germany; 2 Institute of Pathology, Hannover Medical School, Carl-Neuberg-Str. 130625 Hannover, Germany; 3 German Center of Lung Research (DZL), Partnersite BREATH, 306245 Hannover, Germany

**Keywords:** lung, epithelia, interleukin 13, tight junction, UBE2Z, ubiquitin

## Abstract

Interleukin-13 (IL-13) drives symptoms in asthma with high levels of T-helper type 2 cells (T_h_2-cells). Since tight junctions (TJ) constitute the epithelial diffusion barrier, we investigated the effect of IL-13 on TJ in human tracheal epithelial cells. We observed that IL-13 increases paracellular permeability, changes claudin expression pattern and induces intracellular aggregation of the TJ proteins zonlua occludens protein 1, as well as claudins. Furthermore, IL-13 treatment increases expression of ubiquitin conjugating E2 enzyme UBE2Z. Co-localization and proximity ligation assays further showed that ubiquitin and the proteasomal marker PSMA5 co-localize with TJ proteins in IL-13 treated cells, showing that TJ proteins are ubiquitinated following IL-13 exposure. UBE2Z upregulation occurs within the first day after IL-13 exposure. Proteasomal aggregation of ubiquitinated TJ proteins starts three days after IL-13 exposure and transepithelial electrical resistance (TEER) decrease follows the time course of TJ-protein aggregation. Inhibition of JAK/STAT signaling abolishes IL-13 induced effects. Our data suggest that that IL-13 induces ubiquitination and proteasomal aggregation of TJ proteins via JAK/STAT dependent expression of UBE2Z, resulting in opening of TJs. This may contribute to barrier disturbances in pulmonary epithelia and lung damage of patients with inflammatory lung diseases.

## 1. Introduction

Asthma is a complex disease that involves environmental interactions, genetic risk factors and chronic airway inflammation. According to the underlying pathophysiological mechanisms, asthma syndrome was proposed to be subdivided into several endotypes [1]. Depending on the dominating inflammatory response, the endotypes can be grouped in those with low levels of T-helper type 2 cells (T_h_2-cells), the T_h_2 low endotype, and those with high T_h_2-cell levels, described as T_h_2 high endotype [2]. The latter subsumes allergic asthma phenotypes with mainly T_h_2 cell driven symptoms. T_h_2 cells were referred to as T-helper cells that produce and release high amounts of interleukins (IL) -3, -4, -5, -6, -10 and -13 [3]. In particular, IL-13 plays a pivotal role in allergic asthma [4,5]. Novel treatment strategies follow approaches, where IL-13 signaling is inhibited either by immuno-inhibition of IL-13 dependent receptor signaling [6] or by immuno-neutralization of IL-13 itself [6,7,8,9,10]. Both strategies can improve asthma symptoms and reduce risk of exacerbations.

Cigarette smoke is another trigger of pulmonary inflammation, which causes damage and barrier dysfunction [11]. Moreover, cigarette smoke induced inflammatory and metabolic conditions are associated with lung cancer development [12,13]. In this process, IL-13 signaling is considered to be involved via the IL-13 receptor α2 that promotes malignant transformation [14].

IL-13 has multiple effects on pulmonary epithelia. It induces airway hyperresponsiveness [15,16], mucus hypersecretion and airway remodeling [4,17,18,19,20], all of which are the hallmarks of allergic asthma. Additionally, lateral intercellular contacts and tight junctions (TJ) of the pulmonary epithelium are disturbed in patients with allergic asthma [21]. T_h_2 cytokines IL-4 and IL-13 have been found to compromise barrier function of the pulmonary epithelium [22,23,24,25,26].

The barrier function of an epithelium can be described as its ability to limit the diffusion-dependent exchange of solvent and solute between apical and basolateral compartments. Airway epithelial cells are linked by several intercellular protein complexes that connect lateral cell membranes of neighboring cells. TJ form the most apically localized junction complex. They function as the major limiting diffusion barrier as well as a fence that separates the apical and lateral membranes of epithelial cells [27]. TJs are comprised of different claudin (cldn) isoforms and the isoform composition determines the specific functional properties of TJs, specifically their permeability, reflection coefficient and permselectivity [27]. The cldn composition of TJs in the pulmonary epithelium changes along the respiratory tree and adjusts epithelial function to the segment specific requirements [28].

T_h_2 cytokines, particularly IL-4 and IL-13, perturb the protein composition of TJ in pulmonary epithelia via different mechanisms. First, they alter expression levels of several TJ proteins. This has been reported for patients with allergic rhinosinusitis where expression levels of TJ proteins occludin (ocldn) and junctional adhesion molecule A (JAM-A) were reduced and expression of cldn2, a cldn that increases TJ’s permeability in sinonasal epithelial cells, was increased [29]. Similarly, Cldn18 expression levels were significantly reduced in brush samples of human epithelial cells from patients with T_h_2-high asthma endotype that was linked to IL-13 signaling [24]. Second, T_h_2 cytokines decrease protein density at the TJs without affecting expression levels of TJ proteins [24,25].

Many studies revealed that IL-13 is the major effector for perturbation of TJ permeability in T_h_2-high asthma patients [21,22,23,24,25,26]. In addition, breakdown of the pulmonary barrier function is considered to be a hall mark in developing respiratory distress [30]. However, the IL-13 mediated perturbations of TJ are poorly understood. Our study aims at elucidating the effect of prolonged IL-13 exposure on primary human bronchial epithelial cells cultivated at air-liquid interface (ALI) conditions. We demonstrate that IL-13 alters expression levels of cldn8, cldn9 and cldn16, upregulates the E2 ubiquitin conjugating enzyme UBE2Z and induces proteasomal aggregation of TJ proteins together with ubiquitin (UBQ) and UBE2Z. This reduces protein density at the TJ and increases TJ permeability for ions and small molecules and might be an important contributor to patients’ predisposition to asthma exacerbations and subsequent respiratory distress.

## 2. Results

### 2.1. Effect of IL-13 on hTEpC Epithelia

**Figure 1:**
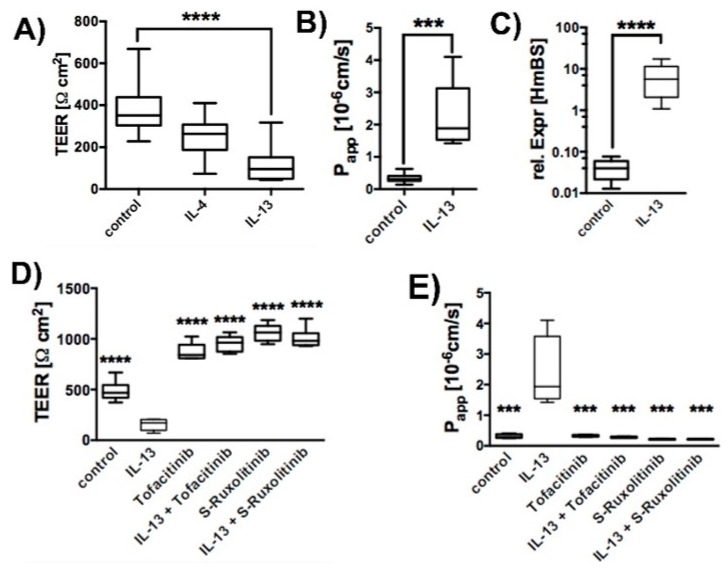
IL-13 increased paracellular permeability. Human tracheal epithelial cells were cultivated at air-liquid interface conditions in the absence (control) and presence of 10 ng/mL IL-13 (IL-13). Box plots summarize data as median values, the boxes represent percentiles, the whiskers indicate the minimum/maximum. (**A**) Response of human tracheal epithelial cells to T_H_2 cytokines IL-4 and IL-13 (10 ng/mL each). Both cytokines reduced TEER. However, TEER reduction by IL-4 did not reach significance (Kruska-Wallis test with Dunn’s correction for multiple testing, *p* = 0.086, IL-4 (*N* = 8) versus control cells (control), *N* = 20). IL-13 reduced TEER significantly (Kruska-Wallis test with Dunn’s correction for multiple testing, *p* < 0.0001, IL-13, N = 17 versus control, *N* = 20). (**B**) IL-13 increases apparent permeability coefficient (P_app_) for the small molecular marker sodium fluorescein (Mann-Whitney test, *p* = 0.0002, *N* = 7). Inhibition of JAK/STAT pathway by Tofacitinib and S-Ruxolitinib abolishes IL-13 induced effects on (**C**) Relative Expression of Muc5ac in human tracheal epithelial cells. Upregulation of mucus production was confirmed by detecting Muc5ac upregulation in IL-13 treated epithelia. (**D**) TEER of control epithelia (control), epithelia cultivated in the presence of IL-13 (IL-13), 2.5 µM Tofacitinib (Tofacitinib) or S- Ruxolitinib (S-Ruxolitinib) and in the presence of IL-13 + Tofactinib or IL-13 + S- Ruxolitinib. (all versus IL-13, ANOVA with Holm-Sidak correction for multiple testing, *p* < 0.0001, *N* = 6) and on (**E**) P_app_ (all versus IL-13, ANOVA with Holm-Sidak correction for multiple testing, *N* = 4, control *p* = 0.0003, Tofacitinib *p* = 0.0003, Tofacitinib + IL-13 *p* = 0.0002, S-Ruxolitinib *p* = 0.0002 and S-Ruxolitinib + IL-13 *p* = 0.0002). *** and **** indicate *p* < 0.001 and *p* < 0.0001, respectively.

Human primary tracheal epithelial cells (hTEpC) were cultivated at air-liquid interface (ALI) for 21 days. Exposure of hTEpC epithelia to 10 ng/mL IL-13 or IL-4 for the entire ALI cultivation time resulted in a transepithelial electrical resistance (TEER) decrease (Figure 1A). Both cytokines are characteristic for T_H_2 high asthma endotype [31]. IL-13 reduced TEER by approximately 85%. The TEER decrease caused by IL-4 was lower and only the effect of IL-13 was statistically significant. Hence, we focused on the epithelial response to IL-13 in all subsequent experiments. The reduction of TEER by IL-13 was in line with an increased apparent permeability coefficient (P_app_) for sodium fluorescein (Figure 1B). This result indicated that IL-13 affects the paracellular diffusion barrier. Increased mucin 5ac (Muc5ac) expression levels (Figure 1C) confirm the biological activity and efficiency of IL-13. Inhibition of JAK/STAT signaling by Tofacitinib [32,33] or S-Ruxolitinib [34,35] (both added at concentration of 2.5 µM) attenuated IL-13 effects on TEER and paracellular permeability (Figure 1D,E). This indicates that IL-13 acts via JAK/STAT signaling on paracellular permeability.

**Figure 2:**
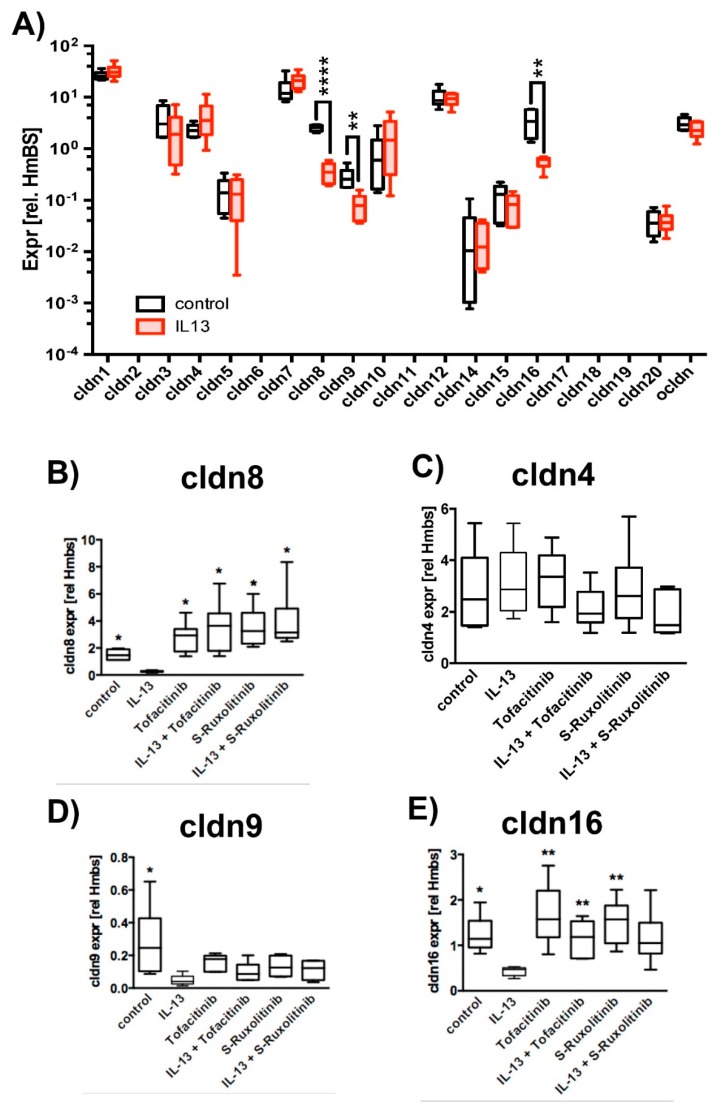
Effect of IL-13 on claudin and occludin expression levels. Human tracheal epithelial cells were cultivated at air-liquid interface conditions. Expression levels were normalized to HmBS. The box plot summarizes data as median values, the boxes represent percentiles, the whiskers indicate minimum/maximum. (**A**) Claudin expression levels in epithelia cultivated in the absence (control) or presence of 10 ng/mL IL-13 (IL-13). Multiple *T*-test, all IL-13 versus control, cldn8 *p* < 0.0001, cldn9 *p* = 0.005 and cldn16 *p* = 0.005, *N* = 6). Transcripts encoding claudins 2, 6, 11, 17, 18 and 19 were not detected. Changes in cldn expression levels were confirmed and the effect of inhibition of JAK/STAT pathway was tested in subsequent independent experiments (control epithelia = control, epithelia cultivated in the presence of IL-13 = IL-13, 2.5 µM Tofacitinib = Tofacitinib or S-Ruxolitinib = S-Ruxolitinib and in the presence of IL-13 = IL-13 + Tofactinib or IL-13 + S- Ruxolitinib, respectively) (**B**) cldn8 expression (Mann Whitney test with Bonferroni correction all versus IL-13 and *N* = 6, control *p* = 0.02, Tofacitinib *p* = 0.02, IL-13 + Tofacitinib *p* = 0.02, Ruxolitinib *p* = 0.02 and IL-13 + Ruxolitinib *p* = 0.02) (**C**) cldn4 expression was not affected neither by IL-13 nor by Tofacitinib or Ruxolitinib. (**D**) cldn9 expression (Mann-Whitney test with Bonferroni correction, *N* = 6, control *p* = 0.04, Tofacitinib *p* = 0.08, IL-13 + Tofacitinib *p* = 0.13, Ruxolitinib *p* = 0.07 and IL-13 + Ruxolitinib *p* = 0.2) and (**E**) cldn16 expression (Mann-Whitney test with Bonferroni correction, *N* = 6, control *p* = 0.02, Tofacitinib *p* = 0.01, IL-13 + Tofacitinib *p* = 0.01, Ruxolitinib *p* = 0.01 and IL-13 + Ruxolitinib *p* = 0.07). *, ** and **** indicate *p* < 0.05, *p* < 0.01 and *p* < 0.0001, respectively.

Paracellular permeability depends on cldn composition of the tight junctions (TJ) [28]. Hence, we investigated the effect of IL-13 on expression levels of claudins and also on occludin (ocldn) as a TJ protein that seals the paracellular pathway. Semi quantitative reverse transcriptase PCR (RT-PCR) experiments revealed a significant reduction of expression levels in IL-13 treated versus control epithelia for cldn8, cldn9 and cldn16 by a factor of 7.0, 3.5 and 6.3, respectively (Figure 2A). Hence, we performed additional experiments to further investigate the effect of IL-13 on cldn8, cldn9, cldn16 expression. Cldn4, although not affected by IL-13, was included in subsequent experiments, since it interacts with cldn8 to form paracellular Cl^−^ pores in TJs [36] (Figure 2B–E). In line with the initial screening, IL-13 treatment reduces cldn8, cldn9 and cldn16 expression levels. Inhibition of JAK/STAT signaling by Tofacitinib [32,33] and S-Ruxolitinib [34,35] abolished the down-regulation of these cldns. Cldn4 expression was not affected neither by IL-13 nor by JAK/STAT inhibition. These experiments confirm that IL-13 specifically changes expression of cldn8, 9 and 16 via JAK/STAT signaling.

**Figure 3:**
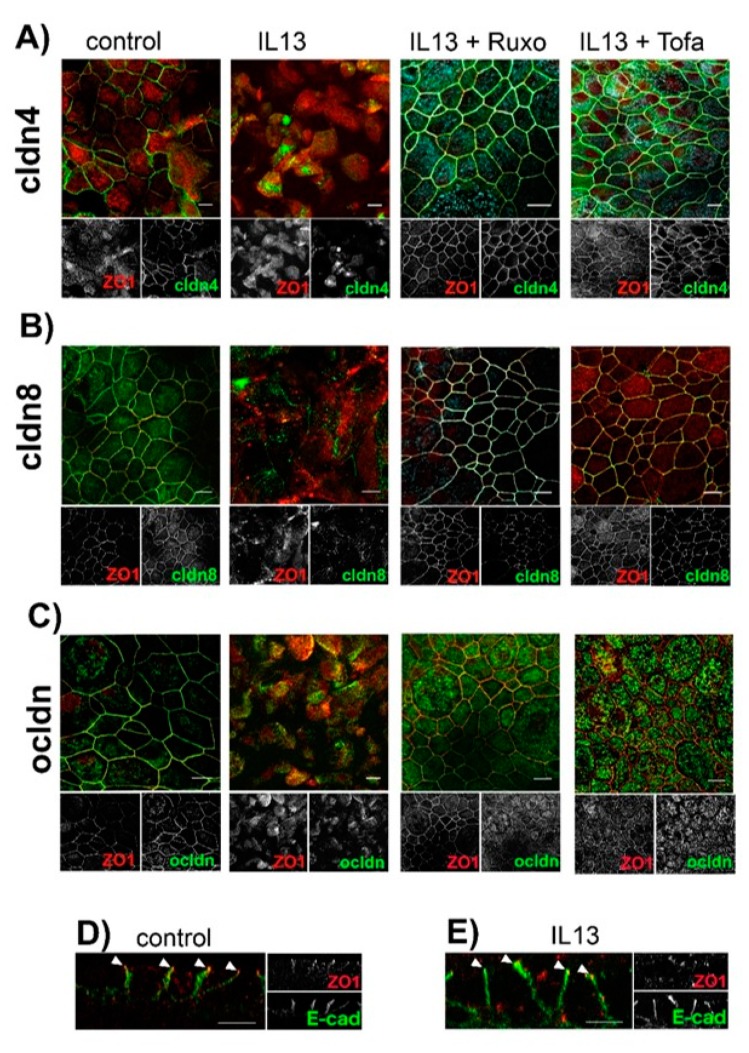
Effect of IL-13 on protein localization at TJ. Human tracheal epithelial cells were cultivated at air-liquid interface conditions. Epithelia were cultivated in the absence of compounds (control), in the presence of IL-13 (IL-13), in the presence of IL-13 + 2.5 µM S-Ruxolitinib (IL-13 + Ruxo) or in the presence of 2.5 µM Tofacitinib (IL-13 + Tofa). Scale bars represent 10 µm. (**A**) Intracellular localization of claudin 4 (cldn4, green channel). Zonula occludens 1 (ZO1, red channel) was used for counterstaining for tight junctions. (**B**) Intracellular localization of claudin 8 (cldn8, green channel). Counterstaining for tight junctions by ZO1 (red channel). (**C**) Intracellular localization of occludin (ocldn, green channel). Counterstaining for tight junctions by ZO1 (red channel). (**D**,**E**) Intracellular localization of E-cadherin (E-cad, green channel) as a marker for lateral adherens junction. ZO1 (red channel) was used for counterstaining of tight junctions.

To further elucidate the mechanisms underlying the IL-13-induced modulation of paracellular permeability, we investigated the cellular localization of TJ proteins (Figure 3A–C). We focused on cldn8, since we recently identified cldn8 as a claudin that regulates protein assembly at the TJs [37]. Cldn4 was chosen as the control, as its expression level is not modulated by neither IL-13 treatment nor inhibitors of JAK/STAT signaling (see above). Finally we investigated ocldn localization, since there is evidence that its localization at TJ in pulmonary epithelia is modulated by cldn8 [37]. In control epithelia, all the investigated TJ proteins colocalized with tight junction marker zonula occludens 1 (ZO1) (Figure 3A–C). Staining for TJ proteins revealed an almost continuous contouring of the cells, indicating their lateral assembly at the TJs. IL-13 treatment resulted in a highly diminished lateral localization of all TJ proteins, including ZO1. In these epithelia, the staining of TJ proteins resulted in a patchy cell contouring that indicates massively impaired protein assembly at the TJ. Moreover, TJ proteins were observed to form intracellular aggregates. This effect was not restricted to IL-13 regulated cldn8 but to all investigated TJ proteins, including ZO1. Inhibition of JAK/STAT signaling using 2.5 µM Tofacitinib [32,33] or 2.5 µM S-Ruxolitinib [34,35] abolished IL-13 induced intracellular localization of TJ proteins. Whereas TJ protein localization was affected by IL-13 treatment, the lateral localization of the adherens junction protein E-cadherin (E-cad) [38] remained almost unaffected by IL-13 (Figure 3D,E). This indicates that the IL-13 effect on cell–cell contacts is restricted to the TJ. Furthermore, IL-13 affects all TJ proteins but not exclusively those proteins, which are regulated at transcriptional levels by IL-13 treatment.

### 2.2. IL-13 Activates the Ubiquitin-Proteasome Pathway

**Figure 4:**
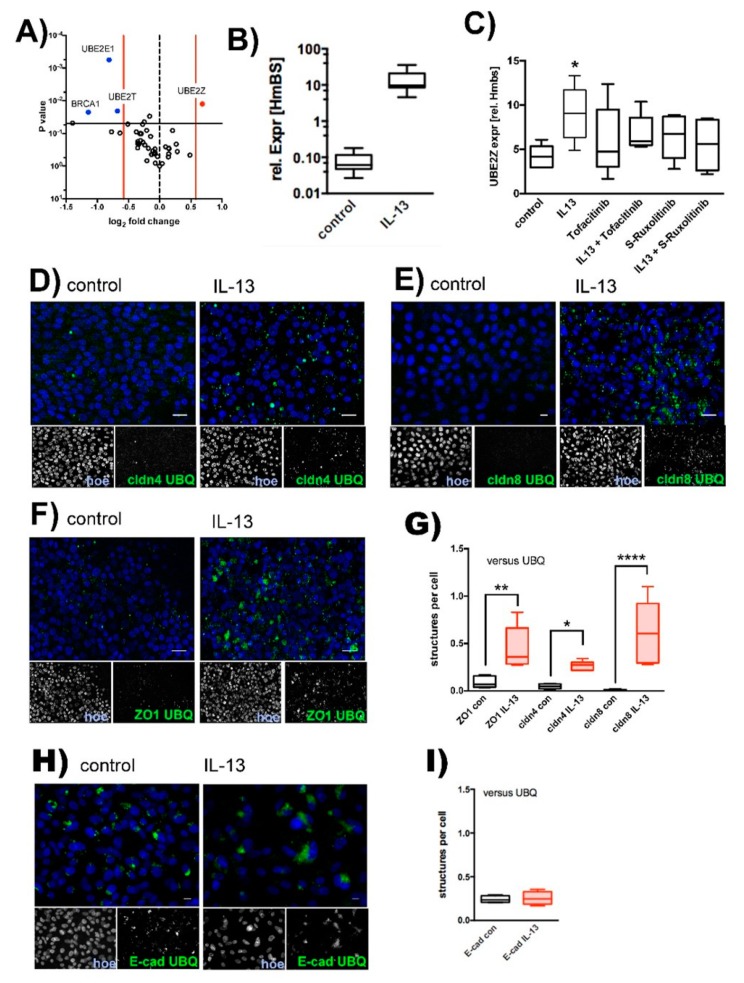
Ubiquitination of TJ proteins. Human tracheal epithelial cells were cultivated at air-liquid interface conditions in the absence (control) and presence of IL-13 (IL-13). (**A**) Screening for ubiquitin activating E1 and ubiquitin conjugating E2 enzyme by semi-quantitative RT-PCR. Volcano plot summarizes changes in expression levels (control vs. IL-13, each *N* = 3). Log2 fold changes are given as mean values. Vertical red lines give threshold of ±1.5 fold change. Horizontal black line gives threshold of *p* = 0.05. Genes that were considered to be up regulated are depicted in red (UBE2Z) and those to be down regulated are given in blue (BRCA1, UBE2T, UBE2E1). (**B**) Upregulation of UBE2Z by IL-13 was confirmed and effect of JAK/STAT pathway inhibition was tested in subsequent independent experiments. Relative expression of UBE2Z to HmBS of: control epithelia (control, *p* = 0.021), epithelia cultivated in the presence of IL-13 (IL-13), 2.5 µM Tofacitinib (Tofacitinib, *p* = 0.035) or S- Ruxolitinib (S-Ruxolitinib, *p* = 0.16) and in the presence of IL-13 + Tofactinib (*p* = 0.16) or IL-13 + S- Ruxolitinib (*p* = 0.09). All versus IL-13 and *N* = 6, ANOVA with Holm-Sidak correction for multiple testing. (**C**) IL-13 stimulation of epithelia used in “B” was confirmed by detecting Muc5ac upregulation (Mann-Whitney test *N* = 6, *p* = 0.004). Ubiquitination of TJ proteins were investigated in proximity ligation assays for (**D**) ubiquitination of claudin 4 (cldn4 UBQ, green channel, (**E**) ubiquitination of claudin 8 (cldn8 UBQ, green channel) and (**F**) ubiquitination of ZO1 (ZO1 UBQ, green channel). Nuclei were counterstained by Hoechst 33342 (blue channels). (**G**) PLA positive structures were identified as green fluorescent particles with an area of 75 µm^2^ and above. Box plot summarizes quantification of PLA positive structures per cell as median, boxes represent percentile and whiskers give minimum/maximum (IL-13 versus control, Anova test with Holm-Sidaks correction for multiple testing, all *N* = 4, ZO1 *p* = 0.0014, cldn4 *p* = 0.03 and cldn8 *p* < 0.0001). (**H**) Ubiquitination of E-cadherin (E-cad UBQ, green channel). (**I**) PLA positive structures per cell summarized as median values, boxes represent percentiles and whiskers provide minimum/maximum values (E-cadherin = E-cad, ubiquitin = UBQ). No difference in PLA signal abundance could be detected in control versus IL-13 treated cells (*N* = 4, Mann-Whitney test *p* > 0.9999). *, ** and **** indicate *p* < 0.05, *p* < 0.01 and *p* < 0.0001, respectively. Scale bars in (**D**–**F**) and (**H**) represent 10 µM.

There is emerging evidence that the ubiquitin-proteasome pathway [39] controls TJ protein turnover and TJ permeability [40,41,42,43,44,45]. The observed localization of TJ proteins at intracellular granules upon IL-13 treatment may hint at the possibility that internalized proteins accumulate at proteasomal compartments. To address the question of whether IL-13 activates the ubiquitin-proteasome pathway, we initially screened for modulation of ubiquitin E1 activating and ubiquitin E2 conjugating enzymes. Semi-quantitative RT-PCR experiments revealed that IL-13 treatment specifically up-regulates expression of the ubiquitin E2 conjugating enzyme, UBE2Z (Figure 4A) 1.6 ± 0.16 fold (mean ± SD, *N* = 3; *T*-test *p* = 0.01). The detected upregulation of Muc5AC expression by IL-13 confirms its efficiency on the tested epithelia (Figure 4B). Subsequent experiments confirmed the upregulation of UBE2Z by IL-13, which was attenuated by inhibition of the JAK/STAT pathway (Figure 4C). The number of cells was limited due to the ALI cultivation on filters and hence limits the applicability of methods like pull down assays. Therefore, to further evaluate ubiquitination of TJ proteins, we performed proximity ligation assays (PLA, Figure 4D–G). In these experiments we investigated if cldn4 (Figure 4C), cldn8 (Figure 4D) and ZO1 (Figure 4E) form protein complexes together with ubiquitin (UBQ) in IL-13 treated cells. Indeed, compared to control cells, segmental analysis (Figure 4F) of cells revealed that IL-13 treatment results in increased number of fluorescent granules that reflect positive PLA signals. This indicates that ubiquitinated proteins and TJ-proteins localize within the same protein complex and suggests that IL-13 induces the ubiquitination of TJ-proteins. Staining for protein complexes that were positive for E-cadherin and UBQ revealed positive PLA signals in control as well as in IL-13 treated cells (Figure 4H). Also, abundance of PLA positive structures did not differ in control versus IL-13 treated cells (Figure 4I). Evidently, E-cad ubiquitination occurs even at basal conditions, presumably as part of protein turnover to maintain proteostasis. This basal E-cad ubiquitination is not affected by IL-13 and agrees with the observation that subcellular E-cad localization remained unaffected by IL-13 treatment shown above, given the results of the immunostaining experiments.

**Figure 5:**
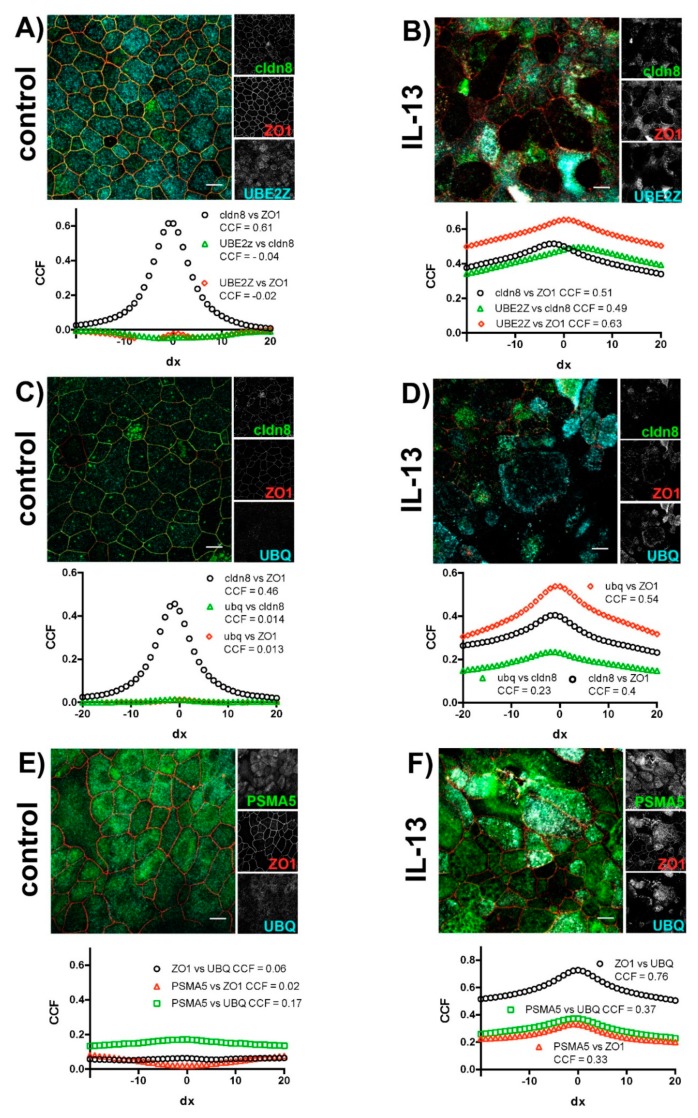
Colocalization of TJ proteins and proteins of the ubiquitin-proteasome machinery. Human tracheal epithelial cells were cultivated at air-liquid interface in the absence (control) and in the presence of IL-13 (IL-13). Images show merged fluorescence channels. Insets at the right give gray scale image for each channel. Van Steensel’s plot below each image gives a cross-correlation coefficient (CCF) plotted against lateral delocalization (dx = 0 to ±20 pixel). Maxima of cross-correlation coefficient plot are given in the legend for each plot. (**A**) Control epithelia and (**B**) IL-13 treated epithelia immune-stained for Claudin 8 (cldn8, green channel), zonula occludens 1 (ZO1, red channel) and UBE2Z (cyan channel). (**C**) Control epithelia and (**D**) IL-13 treated epithelia immune-stained for Claudin 8 (cldn8, green channel), zonula occludens 1 (ZO1, red channel) and Ubiquitin (UBQ, cyan channel). (**E**) Control epithelia and (**F**) IL-13 treated epithelia immune-stained for proteasome subunit α Type 5 (PSMA5, green channel), zonula occludens 1 (ZO1, red channel) and ubiquitin (UBQ, cyan channel). Scale bars in (**A**–**F**) represent 10 µM.

We investigated the intracellular localization of TJ proteins and markers of the ubiquitin-proteasome pathway in subsequent immunocytochemical experiments (Figure 5) and studied their colocalization using van Steensel’s cross-correlation analysis [46]. In control cells, the investigated TJ proteins cldn8 and ZO1 colocalized (Figure 5A,C), resulting in a sharp peak when cross-correlation coefficients were plotted against lateral translocation. Hardly any colocalization could be observed in control cells for the TJ proteins with UBE2Z (Figure 5A) or UBQ (Figure 5C). The exposure of epithelia to IL-13 resulted in an overlapping localization of TJ proteins with UBE2Z (Figure 5B) as well as with UBQ (Figure 5D). In these cases, the van Steensel’s plots revealed increases in the cross-correlation coefficients for all invested proteins. However, the broadening of peak when cross-correlation coefficients are plotted against the lateral translocation reflects the increased and inhomogeneous size of structures where TJ proteins, UBE2Z and UBQ colocalize. No colocalization of ZO1 with the proteasomal marker PSMA5 nor with UBQ or PSMA5 with UBQ could be observed in control epithelia (Figure 5E). In IL-13 treated epithelia (Figure 5F) ZO1 colocalized with UBQ and PSMA5. In these epithelia UBQ colocalizes with PSMA5 as well. Again van Steensel’s plot revealed enlarged non-homogenous size of structures at which colocalization is detected.

### 2.3. Effect of IL-13 on Mature hTEpC Epithelia

**Figure 6:**
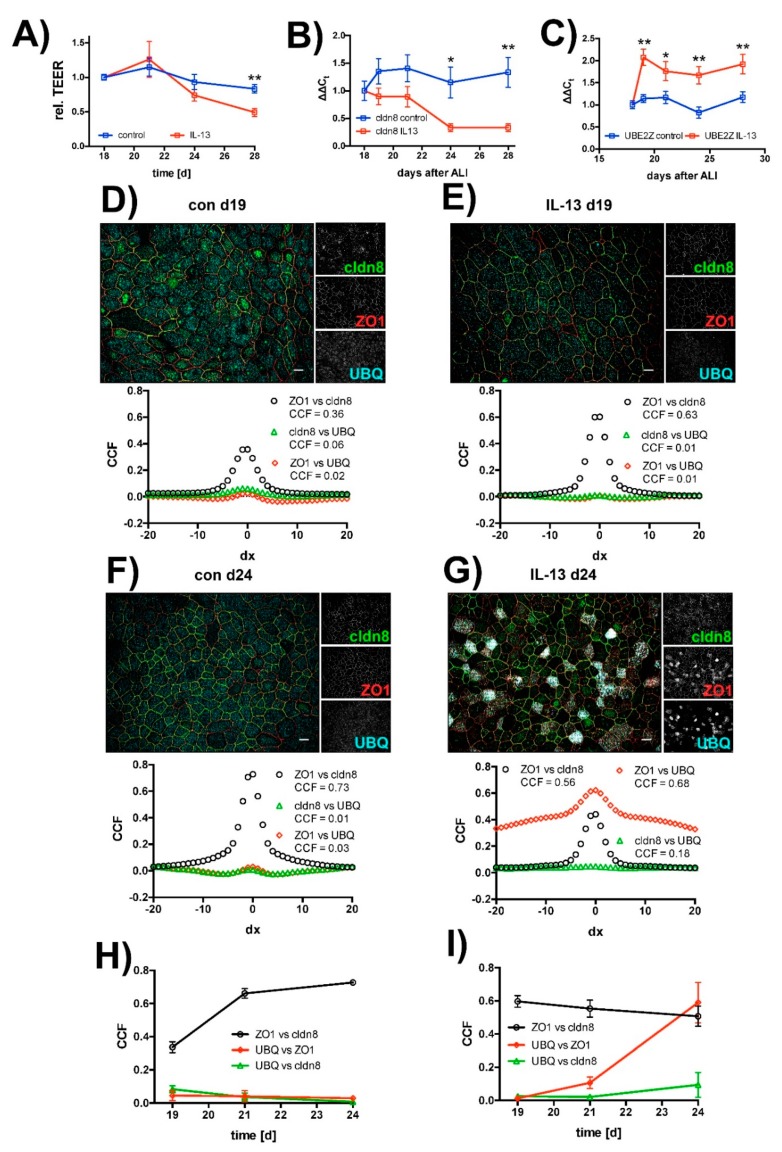
Time course of IL-13 induced effects on human tracheal epithelia. Human tracheal epithelial cells were seeded on Transwell filters and proliferated for 4 days. Afterwards, apical volume was removed and cells were cultivated for additional 18 days at air-liquid interface conditions. On day 18, IL-13 was added to the medium (IL-13). Control cells were not exposed to IL-13 (control). (**A**) Changes of transepithelial electrical resistance (TEER) over time. TEER was normalized to mean TEER measured on day 18 directly before IL-13 exposure and relative TEER was plotted against cultivation time. Rel. TEER decreases from day 21 onwards. Decrease reached statistical significance on day 28. Data points summarize measurements as mean ±SEM (all *N* = 10 to 12, Mann-Whitney test *p* = 0.001). (**B**) Changes of claudin 8 (cldn8) expression levels over time. Changes in cldn8 expression are given as ΔΔ*C*t values relative to the mean expression level on day 18. Data points give mean ΔΔ*C*t ± SEM. Cldn8 expression drops from day 21 onwards. It reached significance on days 24 and 28 (all *N* = 9, Mann-Whitney test *p* = 0.008 and *p* = 0.0002, day 24 and 28, respectively). (**C**) Time course of UBE2Z expression. Changes in UBE2Z expression are given as ΔΔ*C*t values relative to mean expression level on day 18 as mean ΔΔ*C*t ± SEM. UBE2Z expression levels rose directly upon IL-13 exposure an reached significance from day 19 onwards (all *N* = 9, Mann-Whitney test *p* = 0.0012 day 19, *p* = 0.039 day 21, *p* = 0.0027 day 24 and *p* = 0.0019 day 28). Colocalization of tight junction proteins claudin 8 (cldn8, green channel) and zonula occludens 1 (ZO1, red channel) with ubiquitin (UBQ, cyan channel) was analyzed in van Steensel’s plot (below each image). Examples are given for (**D**) control cells on day 19 (con d19) and (**E**) for cells exposed to IL-13 on day 19 (IL-13 d19) as well as (**F**) for control cells on day 24 (con d24) and (**G**) for cells exposed to IL-13 on day 24 (IL-13 d24). Changes of colocalization over time are analyzed by plotting the maximum of cross-correlation coefficients (CCF) from van Steensel’s plot against the cultivation time. Changes of maximum CCF over time (**H**) in control cells and (**I**) in IL-13 treated cells. Black curves give CCF for zonula occludens 1 and claudin 8 (ZO1 vs. cldn8), red curves show CCF for ubiquitin and ZO1 (UBQ vs. ZO1) and green curves show CCF for UBQ and cldn8 (UBQ vs. CLDN8). Data points summarize CCF as mean ± SD all *N* = 3). * and ** indicate *p* < 0.05 and *p* < 0.01, respectively. Scale bars in (**D**–**G**) represent 10 µM.

In order to test if IL-13 also acts on mature epithelia, we added IL-13 at a final concentration of 10 ng/mL to hTEpC epithelia on day 18 after ALI establishment. Measurements were performed on the following days as indicated (Figure 6). In mature hTEpC epithelia, IL-13 reduced TEER from day 21 onwards (Figure 6A). The drop in TEER reached statistical significance on day 28 (control vs. IL-13 treated cells, *N* = 12, Mann-Whitney test *p* = 0.0011). The time course of TEER decrease correlated with time course of cldn8 expression levels, which decreased from day 21 onwards, three days after IL-13 exposure (Figure 6B). The decrease in cldn8 expression levels reached statistical significance from day 24 onwards. The increase in UBE2Z expression levels preceded TEER decrease and occurred already within the first day after IL-13 exposure (Figure 6C). It reached significant levels from day 19 onwards.

Time course of ubiquitination of TJ proteins was investigated in immunocytochemical experiments (Figure 6D–I). In these experiments, we used van Steensel’s cross-correlation analysis [46] to quantify colocalization of ZO1 with cldn8 as well as of UBQ with ZO1 and cldn8. Time course of colocalization was visualized by plotting the maximum Pearson cross correlation coefficient detected from the van Steensel’s plot against cultivation time.

In control cells cross-correlation coefficient for ZO1 and cldn8 colocalization increased from day 19 onwards (Figure 6H). This may reflect gradual tightening of TJ in mature epithelia during ALI cultivation, as it correlates with the slightly increasing TEER (compare Figure 6A). Furthermore, in these control cells no change in cross-correlation over time was observed for ZO1 and cldn8 with UBQ (Figure 6H). 

In case of IL-13 treated epithelia, the cross-correlation coefficient for the colocalization of cldn8 with ZO1 did not change over time. In contrast to that and to control cells, cross-correlation coefficients for UBQ colocalization with ZO1 and cldn8 both increase, starting three days after IL-13 exposure, from day 21 onwards. However, the change in colocalization for cldn8 with UBQ was smaller compared to ZO1 with UBQ (Figure 6I) and most likely reflects a weaker staining efficiency and/or protein abundance of cldn8.

The onset of UBQ colocalization with TJ proteins correlates with onset of TEER decrease. The formation of protein aggregates does not start in all cells simultaneously. Instead, we observed an increasing amount of cells over time that were positive for granules containing tight junction proteins colocalizing with ubiquitin. On day 19 almost no cells were positive for protein aggregates. On day 21, when TEER starts to drop and the cross correlation coefficient starts to rise, 9.3 ± 5.1% of IL-13 treated cells formed aggregates (mean ± SD, *N* = 3). The amount of aggregate containing cells further increased to 23.6 ± 4.6% (mean ± SD, *N* = 3) on day 24. Thus, the time course of protein aggregate formation correlated with time course of epithelial permeability increase. This gives evidence that protein aggregate formation is the TJ damaging step in IL-13 exposed epithelia. These experiments indicate that IL-13 activates the ubiquitin-proteasome pathway that also involves UBE2Z in mature hTEpC epithelia.

### 2.4. Ubiquitination of TJ Proteins in Sinunasal Epithelia

**Figure 7:**
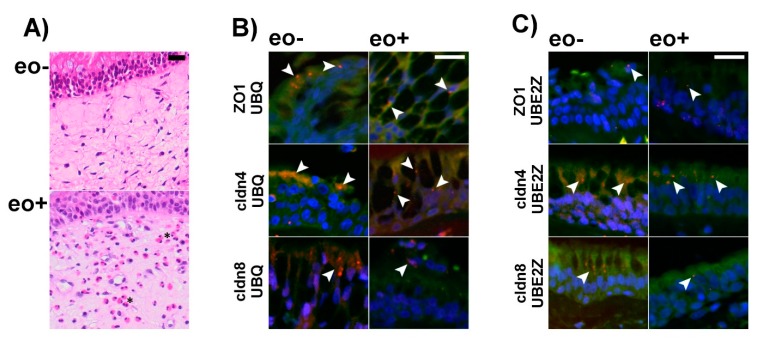
Proximity ligation assays (PLA) were performed on formalin fixed and paraffin embedded tissue from the sinunasal tract containing respiratory type epithelium of patients with and without eosinophilic inflammation. (**A**) shows representative Hematoxilin and Eosin stained images from nasal mucosa with (eo+) and without (eo−) eosinophilic inflammation. Eosinophilic granulocytes can be detected as reddish stained cells in eo+ mucosa (examples are highlighted by asterix) but were not detected in eo- mucosa. For the PLA merge images of nuclear counterstain (blue), autofluorescence (green) and PLA signals (red) are shown. Antibodies specific for ZO1, Claudin 4 and Claudin 8 were combined with antibodies detecting Ubiquitin (**A**) and Ube2Z (**B**). Specific PLA reaction signals (depicted by arrow heads) can be detected in the epithelium under all conditions, suggesting association between ZO1, Claudin 4 and Claudin 8 with Ubiquitin and UBE2Z respectively. Scale bars are 20 µm in panels (**A**–**C**).

To test whether the detected IL-13 induced ubiquitination and proteasomal aggregation also occurs in human airway epithelia, we investigated sinunasal biopsies from patients suffering from chronic inflammation with and without eosinophilia using PLA (Figure 7). These experiments revealed the formation of protein complexes that contain ZO1, cldn4 and cldn8 together with UBQ and UBE2Z. Especially in the absence of bacterial infection, eosinophilic inflammation is a fairly good and specific surrogate for high IL-13 levels [47]. Since measuring IL-13 levels in paraffin embedded tissue biopsies is erroneous, we used eosinophilia as a marker for elevated IL-13 tissue concentrations instead. There was no difference in number of PLA-positive particles detectable in eosinophilic inflammation in comparison to non-eosinophilic inflammation (all as mean ±SD in counts/µm^2^ and *N* = 3 patients. Eosinophilic rhinosinositis: UBQ/cldn4: 0.64 ± 0.18, UBQ/cldn8: 0.78 ± 0.41, UBQ/ZO1: 0.29 ± 0.14 and UBE2Z/cldn4: 0.38 ± 0.02, UBE2Z/cldn8: 0.4 ± 0.19, UBE2Z/ZO1: 0.51 ± 0.26 and non-eosinophilic rhinosinostis: UBQ/cldn4: 0.83 ± 0.28, UBQ/cldn8: 0.56 ± 0.14, UBQ/ZO1: 0.48 ± 0.33 and UBE2Z/cldn4: 0.41 ± 0.06, UBE2Z/cldn8: 1.15 ± 0.89, UBE2Z/ZO1: 0.3 ± 0.05). However, these experiments confirm that UBE2Z dependent ubiquitination is not restricted to in vitro models of airway epithelia but also occurs in vivo.

## 3. Discussion

Early studies on transgenic mice revealed the dominating role of IL-13 in allergic asthma [4]. A more recent study identified IL-13 to modulate TJ permeability via altering claudin expression levels [48]. In accordance with these studies, we demonstrated that long-term exposure of epithelial cells to IL-13 results in perturbed claudin expression pattern and reduced protein densities at the TJs itself. A reduced protein density at the apico-lateral junctions of airway epithelia as a result of T_h_2 cytokine exposure was already described [25,48] and was recently demonstrated to be linked to IL-13/IL-4 induced activation of the JAK/STAT signaling pathway [25], as we also demonstrated herein. Inhibition of either cytokines or their receptors have beneficial effects in patients with allergic asthma (summarized in reference [49]). However, most studies measured the reduction of inflammatory markers or parameters of lung function to quantify the therapeutic effect of IL-4/IL-13 inhibition but not changes in tightness of TJ. Hence these studies cannot be directly compared with the herein reported observations. In inflammatory bowel diseases, IL-13 acts predominantly on transcriptional levels. It seems to increase intestinal epithelial permeability by increasing cldn2 expression levels via activating STAT6, PI3K and MAPK pathways and in addition via reduction of tricellulin expression as a result of JNK, AP1 and ERK1/2 signaling [50]. 

Herein we demonstrate that IL-13 activates the ubiquitin-proteasome system, which involves UBE2Z and finally results in proteasomal aggregation of TJ proteins.

The ubiquitin-proteasome system was formerly described as a system that regulates non-lysosomal protein degradation [39]. It involves the marking of proteins that are targeted for degradation by E1-activating enzymes, E2 conjugases and finally E3 ubiquitin ligases. This sequential process results in a highly regulated and substrate specific degradation of proteins [39]. Later on, investigations revealed that UBQ dependent mechanisms also modulate the abundance of membrane spanning proteins at the plasma membrane and controls their lysosomal as well as non-lysosomal degradation [51,52,53]. Nowadays, it is commonly accepted that the ubiquitin-proteasome system is pivotal in maintaining cellular protein homeostasis via controlling protein turnover, in quality control of protein folding and in guiding of proteins to different subcellular destinations [54]. The ubiquitin-proteasome system has been shown to regulate protein abundance at TJ of cldn1, cldn2 and cldn4 in Madin-Darby Kidney cells (MDCK) cells [55], of cldn5 in endothelial cells [43], of cldn8 in kidney epithelia [40], of cldn4 in rat salivary gland epithelial cells [56], of ocldn in porcine epithelial cells [45], in vascular endothelial cells [57] and in Sertoli cells [42] and of zonula occludens protein 2 in MDCK cells [58]. These studies unequivocally demonstrate that ubiquitin dependent mechanisms are involved in regulating protein composition of TJ and thereby adjust their functional properties. However, the function of ubiquitination of TJ proteins is complex. On one hand, ubiquitination has been shown to label cldn1, cldn2 and ocldn for removal from the TJ via endocytosis and their transport along the endosomal and lysosomal pathway [55,57]. On the other hand, ubiquitination targets cldn5, cldn4 and also ocldn for proteasomal degradation [42,43,45,56]. Thereby, ubiquitination does not only act as a control mechanism to ensure appropriate protein folding or to adjust the protein half-life. It also becomes activated in response to environmental factors to adjust functional properties of TJs, as has been demonstrated for the cAMP dependent cldn4 regulation in Sertoli cells [42] and for ocldn modulation upon muscarinic acetylcholine receptor stimulation [56]. We herein identified IL-13 as another factor that activates the ubiquitin-proteasome system to increase the permeability of TJs. PLA revealed that upon IL-13 exposure, ubiquitinated TJ proteins localized intracellularly rather than at the TJ or anywhere else at the plasma membrane. Sorting and translocation of internalized TJ proteins from endosomal compartments towards the proteasome upon ubiquitination was recently reported for cldn5 [43]. In this study, the authors proposed a derlin1 dependent pathway [43] that presumably involves retrograde transport along endosomal compartments to the endoplasmic reticulum and employs endoplasmic reticulum quality control machinery to translocate the proteins to the proteasome [59,60]. Similar mechanisms could be involved in IL-13 induced proteasomal accumulation of TJ proteins in lung epithelia.

We observed an instantaneous upregulation of the E2 conjugase UBE2Z. The cDNA of UBE2Z was recently cloned and identified to encode an ubiquitin conjugating enzyme [61]. Later reports suggested using UBE2Z to regulate ubiquitin charging of ubiquitin E3 ligases and provide substrate specificity of protein ubiquitination [62,63]. Our experiments strongly support the hypothesis that IL-13 induces ubiquitination and proteasomal accumulation of TJ proteins that are involved in modulation of the paracellular diffusion barrier. Subcellular localization of E-cadherin that forms adherens junctions via homotypic trans-interaction and mediates the dynamic connection of adjacent lateral membranes [64] remained almost unaffected by IL-13. This indicates that lateral inter-cellular adhesion and integrity of epithelial cell layer is not preliminarily disturbed by IL-13. This specificity to TJ is possibly mediated by UBE2Z.

Time course of ubiquitin-mediated regulation of protein abundance at the TJs is reported as a rather fast process, operating within a few hours after stimulation [43,45,56,57,58]. In line with these studies, we observed an instantaneous upregulation of UBE2Z expression, indicating that lung epithelial cells rapidly respond to IL-13 exposure. However, measurable effects on TJ permeability were delayed and correlated with the reduction of protein abundance at the TJ as well as their accumulation at the proteasome. As recently reviewed, aggregation of poly-ubiquitinated proteins at the proteasome occurs in post-ischemic neurons and results from down-regulation of proteasome activity [65]. Indeed, proteasomal hyperactivity reduces aggregation of poly-ubiqutinated proteins [66]. Proteasomal activity depends on intra-cellular ATP [67]. A drop in intra-cellular ATP levels as a result of inflammatory stress conditions (as was introduced herein by IL-13 exposure) in combination with elevated protein transport to the proteasome and proteasomal degradation could exhaust proteasome activity to such an extent that proteins accumulate and aggregate. Impaired proteasomal activity is discussed to affect cellular proteostasis and therefore cause cellular stress and/or damage and also interfere with the location of proteins and the composition of polymeric protein complexes [68]. Such an assumption is in line with a recent study providing evidence that proteasome inhibition in mouse models of inflammatory bowel disease interrupts epithelial barrier function in colonic mucosa [69] and would also explain our observation of the delayed TJ damage as a consequence of impaired intracellular proteostasis.

Because of its distinct function in controlling intra-cellular proteostasis, the ubiquitin proteasome system is believed to play a pivotal role in inflammatory lung diseases and lung injury [70]. Genes involved in protein ubiquitination are up-regulated in the lung tissue of patients with chronic obstructive pulmonary disease (COPD) [71]. This goes in line with the observations that protein ubiquitination is increased in the lung tissue of COPD patients with emphysema [72]. Furthermore, cigarette smoke extract also induces aggregation of poly-ubiquitinated proteins in bronchial epithelial cells [73] and increases protein ubiquitination in lung endothelial cells [74]. These findings underscore the involvement of dysregulation of the ubiquitin proteasome system in the pathogenesis of chronic inflammatory lung diseases like COPD. Our findings are in line with these former observations. They demonstrate that IL-13 results in an incomplete proteasomal degradation of ubiquitinated proteins that coincide with TJ disturbance. They further point towards a yet unknown function of UBE2Z as an effector in IL-13 driven lung inflammation acting on TJs and thereby on paracellular barrier function in pulmonary epithelia. Opening of TJs of the pulmonary epithelium is a hallmark in the pathogenesis of alveolar edema and exudate formation and subsequent respiratory distress [30]. Disturbances of TJs are a predisposing condition for the development of acute respiratory distress syndrome [75] and lung injury for instance following mechanical ventilation [76,77].

Our results gives evidence that UBE2Z dependent ubiquitination of TJ-proteins also occurs in vivo. We used chronic inflammation with eosinophilic granulocytes as surrogate marker for an IL-13 high phenotype [47]. However, there was no significant difference in levels TJ-protein ubiquitination between samples with and without eosinophilic inflammation. Detection of eosinophilia does not measure cytokine concentrations. IL-13 concentrations for eosinophilic non-bacterial lung inflammation was recently measured to reach concentrations of 10.4 pg/mL in the sputum of humans [47] and around 100 ng/mL in bronchoalveolar lavage of IL-13 overexpressing transgenic mice [78]. These measurements may give an estimation of the range of IL-13 tissue levels during eosinophilic lung inflammation. However, it must be considered that eosinophilia reflects chronic inflammation, while a commonly used nasal steroid therapy in patients with chronic rhinosinusitis potentially reduces inflammatory response and cytokine levels. Under these conditions, IL-13 concentrations are probably below the levels that are needed to initiate the observed TJ protein ubiqutination in the herein investigated in vitro model. However, IL-13 tissue levels might increase and become effective to induce the observed TJ protein ubiquitination during exacerbation episodes. Nonetheless, our results demonstrate that TJ protein ubiquitination that involves UBE2Z also occurs in native tissue.

## 4. Materials and Methods

### 4.1. Compounds

IL-4 and IL-13 were obtained from Merck Millipore, Darmstadt, Germany. S-.Ruxolitinib and Tofacitinib, both were obtained from Selleckchem, distributed by Absource Diagnostics, Munich, Germany. If not mentioned otherwise, IL-13 was added at a concentration of 10 ng/mL. S-Ruxolitinib and Tofacitinib was used at concentrations of 2.5 µM each.

### 4.2. Cell Culture

Transwell permeable supports (Polyester membrane diameter 6.5 mm, 0.4 µm pore size, Costar distributed by Omnilab, Bremen, Germany) were overlaid with 100 µL Collagen I and II solution (StemCell Technologies, Cologne, Germany) diluted 1:100 in phosphate buffered Saline (PBS, Gibco distributed by Thermo Fisher, Darmstadt, Germany) and dried over night at room temperature. Dried filters were UV irradiated and stored until use at 4 °C.

Cryo-preserved passage 1 primary human tracheal epithelial cells (Promocell, Heidelberg Germany and Epithelix, Genève Switzerland) were proliferated in T75 flasks (Sarstedt, Nümbrecht, Germany) using Airway Epithelial Growth Medium (Promocell, Heidelberg, Germany) at 37 °C, 5% CO_2_ and 95% humidity. Upon reaching 80% confluence, cells were detached using DetachKit (Promocell, Heidelberg, Germany) and suspended in Airway Epithelial Growth medium. 3.5 × 10^4^ cells in 200 µL Airway Epithelial Growth Medium were seeded onto collagen-coated filters. Filters were placed in 24 well plates filled with 500 µL Airway Epithelial Growth Medium and incubated at 37 °C, 5% CO_2_, 95% humidity for two days. Afterwards, air-liquid interface (ALI) conditions were established by removing the medium from the apical surface and replacing Airway Epithelial Growth Medium by 500 µL of fresh ALI-Medium. ALI-medium contained DMEM-H and LHC basal medium (both from Gibco via Thermo Fisher, Darmstadt, Germany) at a 1:1 ratio and was supplemented with: insulin 0.87 µM, hydrocortisone 0.21 µM, epidermal growth factor 0.5 ng/mL, triiodothyronine 0.01 µM, transferrin 0.125 µM, epinephrine 2.5 µM, bovine pituitary extract 10 µg/mL, bovine serum albumin 0.5 mg/mL (all from Promocell, Heidelberg, Germany), phosphorylethanolamine 0.5 µM, ethanolamine 0.5 µM, zinc sulfate 3 µM, retinoic acid 0.05 µM, ferrous sulfate 1.5 nM, calcium chloride 0.6 µM, magnesium chloride 0.11 µM, sodium selenite 30 µM, manganese chloride 1 µM, sodium silicate 0.5 nM, ammonium molybdate tetrahydrate 1 µM, ammonium metavanadate 5 µM, nickel sulfate 1 µM, tin chloride 0,5 µM (all from Sigma-Aldrich GmbH, Steinheim, Germany), 100 U/mL Penicillin and 100 µg/mL Streptomycin (both from Gibco via Thermo Fisher, Darmstadt, Germany). Medium was replaced every second day. Cells were incubated at 37 °C, 5% CO_2_ and 95% humidity for the time given in the text.

### 4.3. TEER Measurement

Transepithelial electrical resistance (TEER) was measured by impedance spectroscopy using the cellZscope (NanoAnalytics, Münster, Germany). For measurements, the basal electrode was overlaid by 500 µL equilibrated ALI medium. After inserting filters, 100 µL of ALI medium were added to the apical surface and the apical electrode was placed into the apical liquid. The measurements were performed immediately after positioning of apical electrodes., The software package provided with the instrument (NanoAnalytics, Münster, Germany) was used for data acquisition and analysis.

### 4.4. Paracellular Permeability

The paracellular permeability was measured as the apparent permeability coefficient (P_app_) of Na^+^-fluorescein (Sigma-Aldrich GmbH, Steinheim, Germany). Na^+^-fluorescein was added to the basolateral medium at a concentration of 150 µM. The Apical surface was overlaid with 100 µL of isotonic NaCl-solution. After incubation for 30 min at 37 °C, 95% humidity and 5% CO_2_, the Na^+^-fluorescein concentration was measured in the apical solution using an Infinite 200 plate reader with iControl software package (Tecan, Männedorf, Switzerland). P_app_ was calculated according to the equation: P_app_= (c_ap_ × V_ap_)/(c_0_ × t × A) with c_ap_ = Na^+^-fluorescein concentration measured in the apical compartment after incubation, V_ap_ = Volume of isotonic NaCl-solution added to the apical side, C_0_ = Na^+^-fluorescein concentration in the basolateral compartment at time point t = 0, t = incubation time and A = epithelial surface area.

### 4.5. qRT-PCR

Total RNA was isolated from cells grown at ALI conditions in the absence or presence of IL-13, as indicated. Cell lysis was performed using my-Budget RNA Mini Kit (Biobudget, Krefeld, Germany) without prior cell suspension using the lysis buffer provided with the kit. RNA was isolated according to manufacturers’ protocol. First strand synthesis was carried out on 0.6 to 0.8 µg total RNA using the VILO SuperScript cDNA Synthesis Kit and qRT-PCR was performed using SYBR GreenER qPCR Supermix (all from Thermo Fisher, Darmstadt, Germany) according to manufactures’ protocols with Quantitect RT-PCR primer assays (Qiagen, Hilden, Germany). Quantitect primer assays: Cldn1 QT00225764, Cldn2 QT00089481, Cldn3 QT00201376, Cldn4 QT00241073, Cldn5 QT01681232, Cldn6 QT00235193, Cldn7 QT00236061, Cldn8 QT00212268, Cldn9 QT00209482, Cldn10 QT00031101, Cldn11 QT00008085, Cldn12 QT01012186, Cldn14 QT00234731, Cldn15 QT01017723, Cldn16 QT00039655, Cldn17 QT01018507, Cldn18 QT00039550, Cldn19 QT00083475, Cldn20 QT00218057, Occludin QT00081844, Hmbs QT00014462, UBE2Z QT0082516 (all from Qiagen, Hilden, Germany). To screen for transcripts encoding ubiquitin activating enzymes E1 and ubiquitin conjugating enzymes E2 ubiquitination RT^2^ Profiler PCR array and RT^2^ SYBR Green qPCR Mastermix were used (all from Qiagen, Hilden, Germany). RealPlex2 thermocycler with latest version of Realplex data acquisition and analysis software were used (Eppendorf, Hamburg, Germany). PCR cycling protocol: Initial denaturation and Taq activation 95 °C for 2 min, 40 cycles with denaturing 95 °C for 15 s, primer annealing and amplification 60 °C 75 s. PCR products were verified by determining melting curves, final denaturation 95 °C for 15 s, annealing at 60 °C for 15 sec and heat ramping from 60 to 95 °C with a ramp duration of 20 min. Crossing points were determined using the CalPlex method provided with the data acquisition and analysis software. Each target gene expression was determined as a triplet. PCR efficiency, correction for efficiency and relative expression were calculated as previously described [79].

### 4.6. Immunocytochemistry

Epithelial cells cultivated as ALI with and without exposure to IL-13 were washed with ice cold PBS and immediately fixed with ice-cold methanol for 1 min. After methanol removal and washing with PBS, filters and cells were incubated in blocking buffer (PBS with 1% Triton X-100 and 5% BSA) for 5 min at room temperature (RT). After removal of blocking buffer, primary antibody incubation was performed (1 h, RT). Antibodies were diluted in Diluent buffer (PBS with 0.2% Triton X-100 and 0.5% BSA). The following antibodies were used: anti-claudin 4 (ab53156), anti-ZO1 (ab99462), anti-ubiquitin (ab7254), anti-proteasome α-subunit type 5 (PSMA5, ab189855), anti-E-cadherin (ab1416) (all from abcam, Cambridge, UK), anti-claudin 8 (LS-B5505) (Life Span Bioscience, Seattle, WA, USA), anti-UBE2Z (H00065264-M01) (Abnova, Taipei, Taiwan) and anti-occludin (MAB7074) (R&D Systems, Minneapolis, MN, USA). Antibodies were removed by three-times washing with PBS buffer and filters were incubated for 30 min at RT with secondary antibodies diluted in Diluent buffer. Secondary antibodies were: Alexa Fluor 488 donkey anti-rabbit IgG, Alexa Fluor 568 donkey anti-goat IgG and Alexa Fluor 647 donkey anti-mouse IgG (all from Invitrogen, Karlsruhe, Germany). Hoechst 33342 (Invitrogen, Karlsruhe, Germany) was used for staining of the nuclei. Afterwards, filters were washed with PBS and mounted on coverslips using ibidi mounting medium (ibidi, Martinsried, Germany). Images of individually cultivated epithelia were taken from a randomly selected area close to the filter center on an inverted confocal microscope (Leica TCS SP5, Leica, Germany) using a 40× lens (Leica HCX PL APO CS 40× 1.25 oil). Images for the blue (DAPI), green (AlexaFluor 488), red (AlexaFluor 568) and far-red (AlexaFluor 647) channels were taken in sequential mode using appropriate excitation and emission settings. Images represent sections with 90 to 150 cells each. Image analysis was performed using latest version of ImageJ software (NIH, Bethesda, MD, USA) [80]. Colocalization analysis was performed according to the method established by van Steensel [46]. In brief: Cross-correlation of a dual labeling image was calculated by shifting one channel image pixelwise over a distance of ±20 pixel with respect to the other channel. Pearson’s correlation coefficient was calculated and the CCF-value was obtained by plotting Pearson’s correlation coefficient against the translocation of channels, Δx. A maximum of this function at Δx = 0 indicates overlapping fluorescence signals, a minimum of this function at Δx = 0 excludes overlapping fluorescence signals. A randomly distribution of fluorescence signals was indicated by a function without any minima/maxima.

### 4.7. Proximity Ligation Assay

Adjacent proteins were identified by proximity ligation assays (PLA) using the Duolink kit (Sigma Aldrich, Steinheim, Germany) on methanol fixed and permeabilized epithelia (primary antibodies see above). Secondary antibodies: Anti-Mouse MINUS DUO92004, Anti-Rabbit PLUS DUO92002, Goat Plus 82023 (supplied with the Duolink kit). PLA was performed according to the manufacturer’s protocol. IMIC digital microscope (Till Photonics, Muenchen, Germany) equipped with UApo/340 40× /1.35 oil objective (Olympus, Hamburg, Germany) and latest version of Live Acquisition were used for microscopy, (Till Photonics, Muenchen, Germany). Images were taken for each individually cultivated filter from a single randomly selected region close to the filter center. PLA signals were quantified using latest version of ImageJ software [80] after binarization of particles with an area of 75 µm^2^ and above. 

### 4.8. Immunohistochemistry

Formalin-fixed paraffin embedded tissue of sinunasal biopsies with chronic inflammation with and without eosinophilia were selected randomly from the archives of the Institute for Pathology (Hannover Medical School). For evaluation 4 µm thick sections were taken and stained with the proximity ligation assay Duolink (Sigma-Aldrich GmbH, Steinheim, Germany). Primary and secondary antibodies are given above. PLA was performed according to manufacturer’s protocol. Afterwards, the reaction to specimens was embedded with a medium containing DAPI. Representative microscopy images of respiratory epithelia were taken on a Keyence fluorescence microscope (BZ9000) with a 20× magnification lens using the appropriate filter sets to detect DAPI (Excitation 377/50 nm, Beamsplitter 409 nm, Emission 447/60 nm), green (autofluorescence) (Excitation 472/50 nm, Beamsplitter 495 nm, Emission 520/35 nm) and far red (Excitation 628/40 nm, Beamsplitter 660 nm, Emission 692/40 nm) signals. Microscopy images were fused using ImageJ. After linear intensity adjustment, a region of interest containing in-focus and intact respiratory epithelium was marked. PLA signals within this area were manually counted. Recorded values are given as counts per area. All experiments were performed with approval of the ethics committee of the Hannover Medical School (Project no. 2699-2015, approved April 2015).

### 4.9. Statistical Analysis

GraphPad Prims6 software (GraphPad, La Jolla, CA, USA) was used for statistical analysis (tests are given with the text). For each experiment cells were obtained from 2 to 4 individual donors. Number of experiments (N) gives number of individual cultivated filters. Differences were considered to be significant if calculated *p*-values were *p* ≤ 0.05. Significance levels are given within the diagrams as: 0.05 ≥ *p* > 0.01 = *, 0.01 ≥ *p* > 0.001 = **, 0.001 ≥.001 = *, *p*-levels are given as 0.00001 = ****. Used test methods and exact *p*-values are given within the figure legends.

## Abbreviations

ALI	air-liquid interface
CCF	cross-correlation coefficient
cldn	claudin
E-cad	E-cadherin
hTEpC	human tracheal epithelial cells
IL	interleukin
JAK	janus kinase
JAM-A	junctional adhesion molecule A
MDCK	Madin-Darby kidney cells
muc5ac	mucin 5AC
ocldn	ocludin
P_app_	apparent permeability coefficient
PLA	proximity ligation assay
PSMA5	proteasomal subunit alpha type 5
RT-PCR	reverse transcriptase PCR
STAT	signal transducer and activator of transcription
TEER	transepithelial electrical resistance
T_h_2	T-helper cell type 2
TJ	tight junction
UBE2Z	ubiquitin conjugating enzyme 2Z
UBQ	ubiquitin
ZO1	zonula occludens 1 protein
